# Persistent 2-for-1 Atrioventricular Node Anterograde Conduction During a Supraventricular Tachycardia

**DOI:** 10.1016/j.jaccas.2022.03.003

**Published:** 2022-05-18

**Authors:** Ankur Shah, Brad A. Clark, Saarik Gupta, Jasen L. Gilge, Asim S. Ahmed, Parin J. Patel, Leonard A. Steinberg, Benzy J. Padanilam

**Affiliations:** a Division of Cardiology, Department of Internal Medicine, Ascension St Vincent, Indianapolis, Indiana, USA; b Division of Cardiology, Ascension Sacred Heart Pensacola, Pensacola, Florida, USA

**Keywords:** atrioventricular nodal nonre-entrant tachycardia, double fire, supraventricular tachycardia, 2-for-1, AT, atrial tachycardia, AVNRT, atrioventricular nodal re-entrant tachycardia, AVRT, atrioventricular re-entrant tachycardia, FP, fast pathway, IP, intermediate pathway, SP, slow pathway, SVT, supraventricular tachycardia, TFOR, 2-for-1 response

## Abstract

We present a case of persistent dual AV node conduction during AV node reentry tachycardia as a new clinical manifestation of 2-for-1 AV node conduction. The interpretation of the complex physiology ponders the possibility of an accessory pathway mediated atrioventricular reentry existing with more ventricular than atrial events.

## Central Illustration

**Figure:**
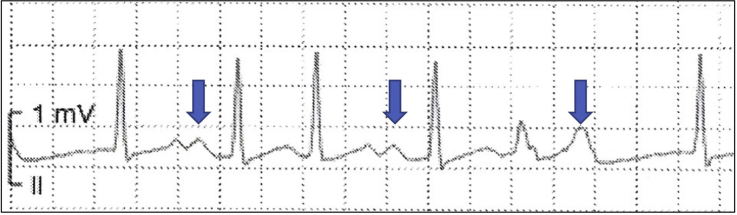

## History of Presentation

**Figure 1:**
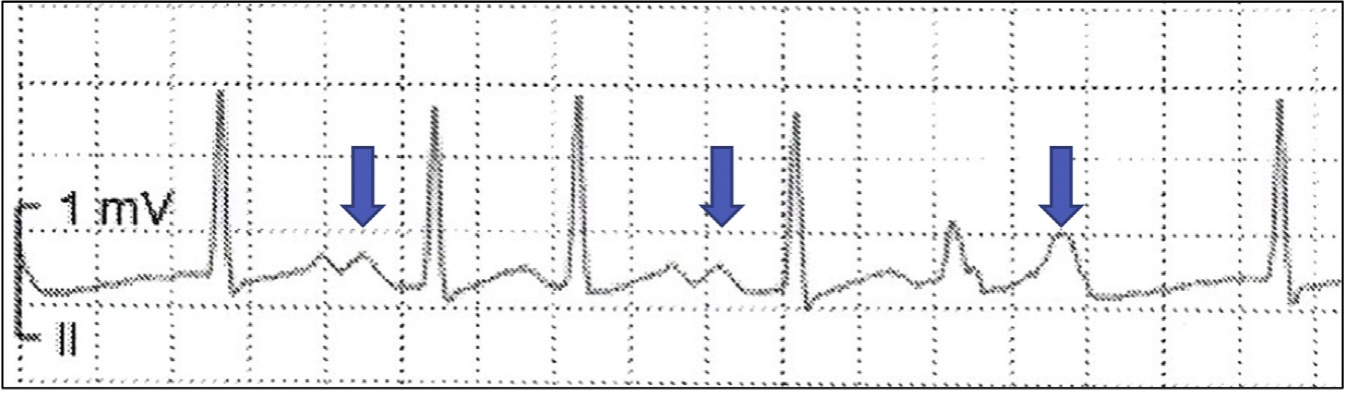
Telemetry Strip Telemetry recording showing 2-for-1 response. The first and second P waves **(blue arrows)** are followed by 2 QRS complexes resulting from simultaneous conduction down the fast and slow AV node pathways. The third P-wave falls on top of the T-wave, blocks in the fast pathway, and conducts only down the slow pathway. Note the aberrant QRS on the fifth complex.

A 33-year-old woman presented to the emergency room with rapid palpitations. Telemetry monitoring showed sinus rhythm with 2 QRS complexes after 1 P-wave suggesting atrioventricular (AV) nodal 2-for-1 response (TFOR) (Figure 1). Medical therapy including intravenous diltiazem was ineffective, and the patient underwent electrophysiology study.

**Learning Objectives**

• To recognize a new clinical manifestation of 2-for-1 atrioventricular node conduction.
• To understand the complex physiology when persistent 2-for-1 conduction occurs during a supraventricular tachycardia.
• To ponder the question whether atrioventricular re-entry can exist with more ventricular than atrial events.

## Physical Examination

The result of physical examination was unremarkable aside from the irregular pulse.

## Medical History

The patient had no significant medical history.

## Investigations

**Figure 2:**
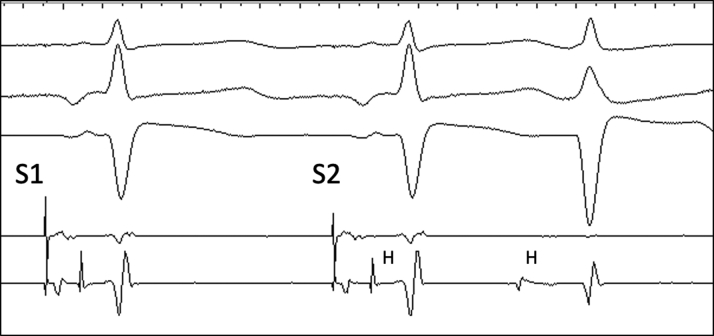
2-for-1 Response With Atrial Extrastimulus Drive train (S1) is 900 ms and extrastimulus (S2) 730 ms. Note the aberrant QRS and HV interval prolongation during 2-for-1 response. H = His signal.

**Figure 3:**
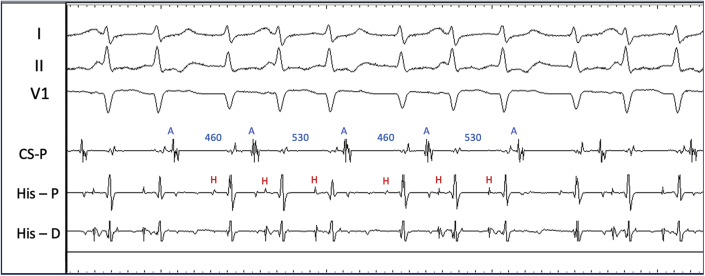
Irregular Supraventricular Tachycardia Note the alternating atrial cycle lengths and 2 QRS complexes within the longer atrial cycles and 1 QRS complex within the shorter atrial cycle. A = atrial electrogram; H = His electrogram.

During electrophysiology study, the baseline sinus cycle length was 1104 ms, AH interval was 96 ms, and HV interval was 54 ms. Dual AV nodal physiology was observed with atrial extrastimulus pacing. During atrial incremental and extrastimulus pacing, there was apparent TFOR conduction (Figure 2), but no sustained tachycardia was induced. Ventriculoatrial conduction was absent at baseline but 1:1 up to pacing cycle length 450 ms during isoproterenol infusion. On isoproterenol 15 μg/min, a regularly irregular supraventricular tachycardia (SVT) with a 2:3 ratio between atrial and ventricular activity was induced (Figure 3). The atrial and ventricular rhythms exhibited alternating cycle lengths. Atrial activation was concentric. Premature ventricular complexes (PVCs) during the rhythm did not perturb atrial activation.

## Differential Diagnosis

The complex electrogram patterns (Figure 3) had more ventricular events than atrial events. The atrial and ventricular cycle lengths alternated but were not in unison, and the atrial activation was followed by either 1 or 2 ventricular events. The longer atrial cycles encompassed 2 QRS complexes, and the shorter atrial cycles encompassed a single QRS complex. By established criteria, an SVT with more ventricular events than atrial events excludes atrioventricular re-entrant tachycardia (AVRT) and atrial tachycardia (AT), leaving atrioventricular nodal re-entrant tachycardia (AVNRT), nodoventricular re-entry, or junctional tachycardia as the only possible mechanisms. However, sustained TFOR during SVT may present a rare exception to these general rules, and AVRT and AT still need to be considered.

### Atypical AVNRT with anterograde TFOR

**Figure 4:**
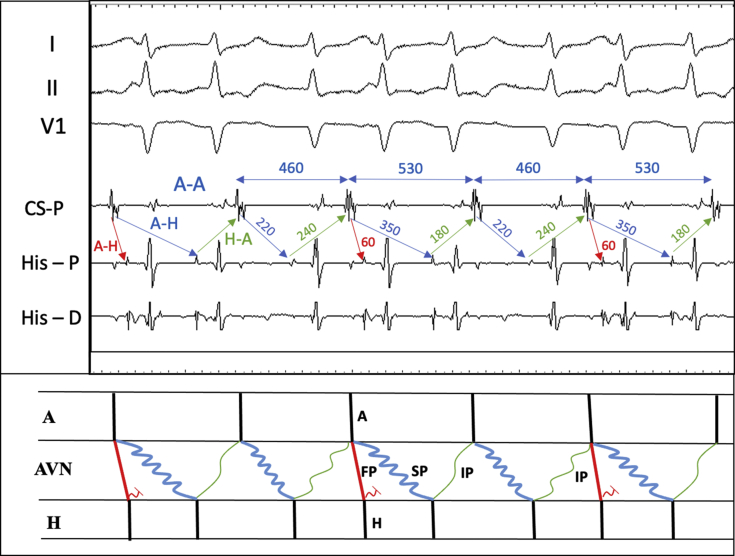
Atrioventricular Node Reentry With 2-for-1 Conduction Inverted P waves are seen on surface lead II at the end of 2-for-1 segment preceding single fire segments. Ladder diagram at the bottom models maintenance of re-entry with ongoing 2-for-1 conduction with anterograde conduction over fast and slow pathways and retrograde conduction over an intermediate pathway. FP = fast pathway; SP = slow pathway; IP = intermediate pathway; A = atrial electrogram; AVN = atrioventricular node; H = His electrogram; CS-P = coronary sinus proximal; RV = right ventricular; P = proximal; D = distal.

This mechanism requires the presence of an intermediate AV nodal pathway (IP) in addition to the AV node fast pathway (FP) and slow pathway (SP) (Figure 4). TFOR occurs on alternate cycles. In atrial cycles with a single ventricular complex, the atrial beat conducts over the AV node SP and returns to the atrium over an IP. Because SP conduction is relatively short (AH 220 ms), retrograde IP conduction decrements resulting in a relatively long HA interval of 240 ms. The next atrial beat conducts antegrade over both the FP and the SP—a TFOR. Conduction over the FP does not participate in re-entry but conceals into the SP to further delay conduction (AH 350 ms). Whereas the absence of retrograde conduction from FP to SP is a requirement for TFOR, concealment resulting in conduction delay may be possible. The re-entrant circuit then returns to the atrium over the IP to complete the circuit. Because of a more prolonged SP conduction, the IP has more recovery time and conducts more rapidly than the previous beat (HA 180 ms). An alternative explanation for the longer SP AH interval during TFOR could be the shorter AA interval preceding it. However, here, a different mechanism becomes necessary to explain why the FP blocks when the preceding AA interval is longer.

Why do TFORs occur only on every other beat? We believe there is retrograde concealment into the FP after each SP activation. The shorter HA interval after the TFOR does not allow for FP recovery, whereas the long HA interval on the alternate beats provides enough time for the FP to recover. Thus, although all the findings are consistent with AVNRT, the diagnosis is one of exclusion, and further deliberation to exclude other arrhythmias is warranted.

### AVRT with anterograde TFOR

The lack of a 1:1 AV relationship typically excludes AVRT, but in the presence of TFORs, an exception may be possible. In this case, AVRT could be mediated by a decrementally conducting accessory pathway. During TFOR, the anterograde FP response finds the retrograde AP refractory, but the anterograde SP response leads to a later ventricular activation that finds AP receptive for retrograde conduction and completes the AVRT circuit. Because the decrementally conducting AP has had more recovery time from the longer AH, retrograde conduction occurs more rapidly, resulting in the relatively shorter HA interval. In atrial cycles with single ventricular responses, the AH is relatively shorter, and the AP conducts retrogradely, with further decrement, resulting in the longer HA interval. The possibility of AVRT as a potential diagnosis in this case is intriguing because it goes against the classic dogma of AVRT requiring a 1:1 atrioventricular relationship.

### Nodoventricular reentry

Here the retrograde conduction occurs via an AP that inserts into the AV node, and ventriculoatrial block occurs from the AV node to the atrium at a 3:2 ratio.

### At with TFOR

With negative surface P waves on the inferior leads and the low to high atrial activation sequence (not shown in the figure), a low AT focus may be considered. However, alternating atrial cycle lengths with coincident changes in AH intervals make it less likely.

### Junctional tachycardia without TFOR

The arrhythmia could also be explained by junctional tachycardia. In this scenario, there are no TFORs. In the arrhythmia noted during electrophysiology study, the regularly irregular pattern could be explained by exit block from a junctional focus.

## Management

**Figure 5:**
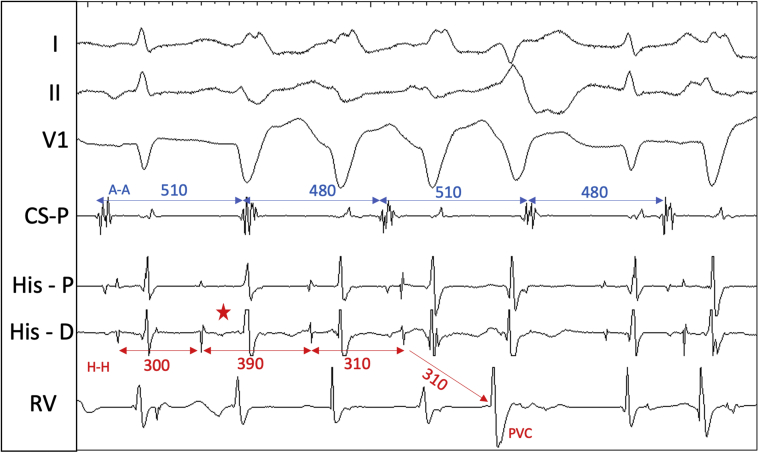
Premature Ventricular Complex The A-A interval remains unchanged after PVC and HV interval prolongation excluding atrioventricular re-entry. Note the marked HV prolongation with left bundle branch aberration **(star)**.

Although we could not entirely exclude many of the other arrhythmia mechanisms in this patient, the diagnosis of AVNRT was thought to be most likely. Whereas AVNRT could be explained by TFORs alone, both AT and AVRT required the presence of TFORs and a second unusual phenomenon. In the case of AT, the observations required the presence of TFORs plus an irregular atrial arrhythmia. Similarly, AVRT required the presence of TFORs plus a decrementally conducting septal AP. Furthermore, PVCs did not perturb the atrial activation, and HV prolongation did not affect atrial cycle lengths (Figure 5). Nodoventricular re-entry and junctional tachycardia did not require any of the above unusual findings but seemed less likely in the presence of reproducible TFOR with atrial extrastimulus pacing. Adenosine testing was not performed, and its response may overlap between the rhythms above. The retrograde atrial activation is earlier at the proximal coronary sinus compared with His, a feature of atypical AVNRT. A standard radiofrequency ablation of AV node SP was undertaken because there was evidence for anterograde SP in addition to retrograde slow/intermediate pathway. After ablation there was elimination of all SP conduction and arrhythmias.

## Follow-Up

There were no recurrent clinical arrhythmias at the 1-year follow-up visit.

## Discussion

To the best of our knowledge, the persistent TFOR during SVT has not been reported before. Multiple manifestations of TFORs have been described in the literature, including premature beats, a dual AV nodal non-re-entrant tachycardia, irregular rhythms resembling atrial fibrillation and heart block.^1^^,^^2^ It has also been described in response to premature atrial complexes during AVNRT.^3^ This case adds to the unusual manifestations of TFOR and gives us extreme physiologic possibilities to ponder the question whether AVRT can exist with more ventricular than atrial activations.

## Conclusions

Persistent dual AV node conduction during SVT is a new clinical manifestation of TFOR and can lead to challenging complex physiology.

## Funding Support and Author Disclosures

The authors have reported that they have no relationships relevant to the contents of this paper to disclose.
